# Atrial Fibrillation Risk in Relation to the Clinical Staging of Gastric Cancer: A Nationwide Population-Based Cohort Study

**DOI:** 10.3390/cancers17122054

**Published:** 2025-06-19

**Authors:** Mi Jin Oh, Yoon Jin Choi, Jin-Hyung Jung, Seunghan Lee, Kyungdo Han, Soo-Jeong Cho

**Affiliations:** 1 Department of Internal Medicine and Liver Research Institute, Seoul National University College of Medicine, Seoul 03080, Republic of Korea; 2 Center for Gastric Cancer, National Cancer Center, Goyang 10408, Republic of Korea; 3 Samsung Biomedical Research Institute, Sungkyunkwan University School of Medicine, Suwon 16419, Republic of Korea; 4 Department of Statistics and Actuarial Science, Soongsil University, Seoul 06978, Republic of Korea

**Keywords:** gastric cancer, atrial fibrillation, SEER program, epidemiology, risk factors

## Abstract

Background/Objectives: Patients with gastric cancer (GC) have an elevated risk of atrial fibrillation (AF) and cardiovascular mortality, compared with the general population. However, the effect of the cancer stage on the development of AF remains unclear. This study aimed to evaluate the relationship between the risk of AF and GC stage based on the Surveillance, Epidemiology, and End Results (SEER) stage classifications. Methods: This retrospective population-based cohort study enrolled patients diagnosed with GC between 2012 and 2019, using anonymized data from the Cancer Public Library Database of South Korea. Patients were followed up until 2020. The risk of AF was assessed in relation to the SEER stage of GC (localized, regional, distant) using adjusted hazard ratios (aHRs) with 95% confidence intervals (CIs). Subgroup analyses were performed according to age, sex, year of diagnosis, and comorbidities. Results: Of the 211,500 patients enrolled in this study, 7266 were diagnosed with AF during follow-up. The risk of AF increased progressively with cancer stage, with aHRs of 2.00 (95% CI 1.81–2.22) for the distant stage and 1.32 (95% 1.25–1.41) for the regional stage, compared with the localized stage. Subgroup analyses showed a consistent association between advanced cancer stage and a higher AF risk; the association was stronger in the younger, female, and non-hypertensive subgroups. Conclusions: The risk of AF in patients with GC is associated with the initial stage, highlighting the need for the closer monitoring and management of AF to improve the survival of patients with advanced-stage GC.

## Simple Summary

While the elevated risk of atrial fibrillation (AF) in patients diagnosed with cancer is well established, the effect of cancer stage on the risk of AF is unclear. A nationwide cohort study in Korea revealed a progressive increase in AF risk according to the clinical stage of gastric cancer, especially in those without conventional risk factors for AF. This highlights the need for closer monitoring and management of AF to improve the survival of patients with advanced-stage gastric cancer.

## 1. Introduction

Gastric cancer (GC) is a leading malignancy worldwide and ranks fifth in terms of incidence and mortality [1]. In South Korea, gastric cancer is the most prevalent cancer and fourth most commonly diagnosed cancer, with an incidence rate of 57.2 per 100,000 in 2021 [2]. As survival rates among gastric cancer patients have improved over recent decades, there is growing interest in the long-term management of gastric cancer survivors [3]. Recent studies have shown that patients with gastric cancer have an increased risk of cardiovascular diseases, including atrial fibrillation (AF), and a higher cardiovascular mortality compared to the general population [4,5].

The association between cancer and AF has been demonstrated not only in gastric cancer but also in other cancer types, as shown in previous studies [6,7,8,9,10,11]. Numerous shared risk factors—such as advanced age, male sex, obesity, and smoking—as well as overlapping pathophysiological mechanisms, including heightened inflammation and neurohormonal dysregulation, may explain this relationship. Additionally, cancer treatment-related complications, such as those arising from chemotherapy or radiotherapy, may further contribute to the development of AF [10,11]. However, no data are currently available regarding the risk of AF in relation to gastric cancer stage at the time of diagnosis. Given that shared pathophysiological mechanisms and cancer-associated complications may promote AF’s development, we hypothesized that patients diagnosed with gastric cancer at an advanced stage may have a higher risk of AF. Therefore, this study aimed to investigate the risk of AF according to the clinical stage at the time of gastric cancer diagnosis.

## 2. Materials and Methods

### 2.1. Data Source and Study Population

**Figure 1:**
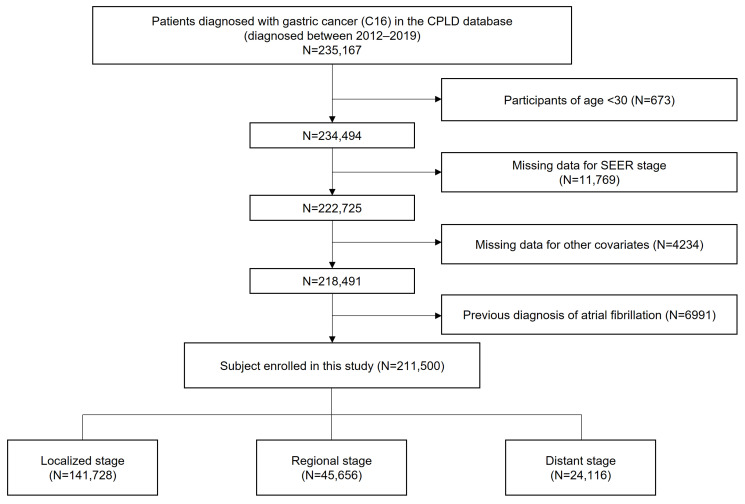
Flowchart showing the enrollment process.

This study utilized anonymized data from the Cancer Public Library Database of South Korea, a comprehensive database of patients newly diagnosed with cancer between 2012 and 2019 [12]. Patients diagnosed with GC were identified using the International Classification of Diseases for Oncology, 3rd edition (ICD-O-3) code C16; subsequently, a cohort of 235,167 patients with GC was constructed. Those aged under 30 years (N = 673) and those with missing data for SEER stage (N = 11,769) and other covariates (N = 4234) were excluded. Among these patients, those who had been diagnosed with AF prior to cancer diagnosis, identified by the ICD-10 code I48, were excluded (N = 6991). Ultimately, a total of 211,500 patients were analyzed. (Figure 1) The index date was defined as the date of GC diagnosis, and the cohort was followed up until the diagnosis of AF, death, or the end of the study period. To ensure a minimum of one year of follow-up for all patients and to minimize follow-up bias, the study period was concluded on 31 December 2020.

This study was exempted from review by the Institutional Review Board of Seoul National University, South Korea (IRB No. 2411-121-1590), and the requirement for informed consent was waived owing to the retrospective design of the study.

### 2.2. Definitions

GC staging was based on the Surveillance, Epidemiology, and End Results Program (SEER) system, classified as localized, regional, or distant. Data on the initial treatments performed within 4 months of diagnosis, including surgery, chemotherapy, radiotherapy, and immunotherapy, was collected. Comorbidities were identified as described in Supplementary Table S1. The primary outcome was a diagnosis of AF, identified using the International Classification of Diseases, 10th revision (ICD-10) code I48, either as a hospitalization diagnosis or confirmed on at least two occasions in outpatient clinics.

### 2.3. Statistical Analysis

Continuous variables were represented as mean ± standard deviation and compared using analysis of variance, whereas categorical variables were expressed as proportions and compared using the chi-square test. AF risk according to the GC SEER stage was calculated using five multivariable Cox proportional hazard models. Model 1 was unadjusted. Model 2 was adjusted for sex and age. Model 3 was additionally adjusted for income and residential area on top of Model 2. Model 4 was additionally adjusted for history of diabetes mellitus, hypertension, and dyslipidemia on top of Model 3. Model 5 was additionally adjusted for initial therapy on top of Model 4. The cumulative incidence of AF was illustrated using Kaplan–Meier curves and compared using the log-rank test. Subgroup analyses were performed based on age, sex, year of diagnosis, and comorbidities.

Statistical significance was set at a two-sided *p*-value < 0.05. All statistical analyses were performed using SAS version 9.4 (SAS Institute, Cary, NC, USA) and R software 4.4.1 for Windows.

## 3. Results

### 3.1. Baseline Characteristics

The baseline characteristics of the study population according to the SEER summary stage at diagnosis are summarized in Table 1. Of the 211,500 patients analyzed, 141,728 were in the localized stage, 45,656 in the regional stage, and 24,116 in the distant stage at diagnosis. Compared to the advanced stages, patients in the localized stage showed a stronger male predominancy and tended to be younger, predominantly in the age between 40 and 64. Most patients in the localized and regional stage received surgery as an initial treatment, while most patients in the distant stage initially received chemotherapy. Patients in the localized stage showed a higher prevalence of hypertension and dyslipidemia compared to the regional and distant stage. The mean follow-up duration of the study population was 3.89 ± 2.58 years, with a significantly shorter follow-up duration in advanced stages (Table 1).

### 3.2. Risk of Atrial Fibrillation According to the SEER Stage at Diagnosis of Gastric Cancer

During the follow-up period, 4765 patients, 1754 patients, and 747 patients in the localized, regional, distant stages, respectively, were diagnosed with AF, with incidence rates of 7.47, 11.06 and 27.73 per 1000 person-years, respectively (Table 2).

**Figure 2:**
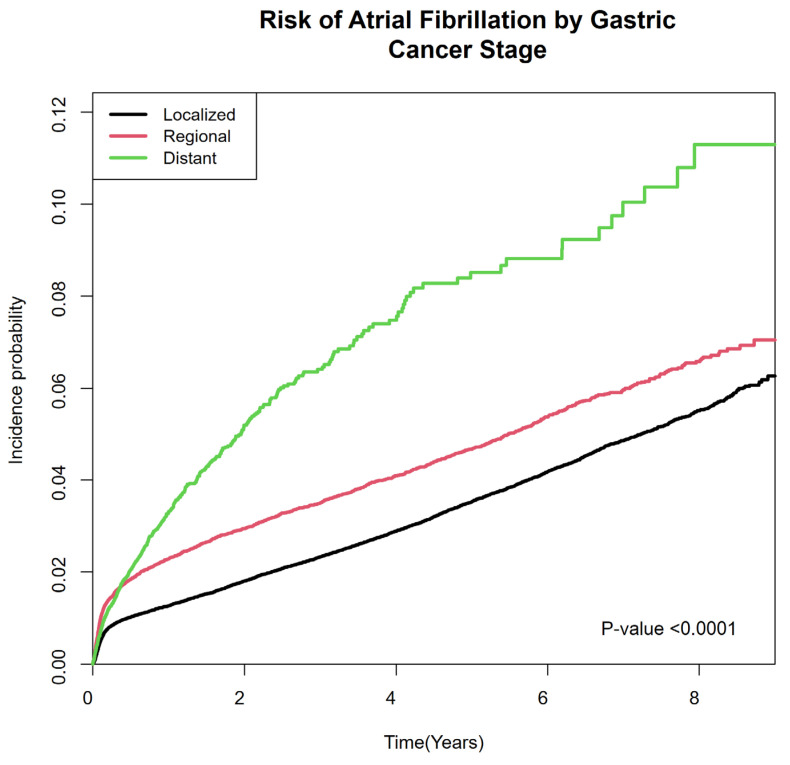
Cumulative incidence of atrial fibrillation in gastric cancer patients according to SEER stage. SEER: Surveillance, Epidemiology, and End Results Program.

In all five models, the risk of developing AF was progressively higher in more advanced stages. In Model 5 which was adjusted for age, sex, income, residence, comorbidities, and initial therapy, the adjusted hazard ratio was 1.32 (95% confidence interval [CI] 1.25–1.41) in the regional stage and 2.00 (95% CI 1.81–2.22) in the distant stage compared with the localized stage. (Table 2) The cumulative incidence of AF also increased progressively according to GC stage (Figure 2).

### 3.3. Subgroup Analysis

Subgroup analyses revealed that the risk of AF was consistently higher in patients diagnosed at a more advanced stage, regardless of age, sex, year of diagnosis, or comorbidities (Table 3). However, a significantly stronger correlation was observed between the GC stage and risk of AF in younger, female, and non-hypertensive subgroups. Additionally, patients diagnosed more recently (in 2016–2019) showed a significantly stronger association between GC stage and AF risk, compared with those diagnosed earlier (in 2012–2015).

## 4. Discussion

An elevated risk of AF in patients with various cancers has been demonstrated in multiple cohort studies and meta-analyses [6,7,8,9,10]. With regard to cancer stage, a meta-analysis investigating the incidence of AF in breast cancer patients found no significant difference between early-stage and metastatic disease [13]. In contrast, a cohort study using the SEER–Medicare linked database reported that a higher SEER stage at diagnosis was associated with an increased risk of AF, showing conflicting results [14]. In the present study, the initial clinical stage of gastric cancer was significantly associated with a risk of AF. This finding suggests that an increased tumor burden or the systemic spread of cancer in advanced-stage gastric cancer is associated with the risk AF. This link between tumor burden and AF aligns with the proposed mechanistic pathway linking cancer and AF, in which heightened systemic inflammation driven by tumor-derived cytokines is thought to play a central role in the development of AF among patients with gastric cancer.

Previous studies have speculated that the elevated risk of AF in patients with cancer may be attributed to the shared risk factors between GC and AF. However, in this study, patients without conventional risk factors for AF, including younger, female, non-hypertensive patients, showed a stronger correlation between cancer stage and AF. One possible explanation is that individuals with conventional AF risk factors, including older, male, hypertensive populations, may already have an elevated baseline risk of AF, regardless of cancer stage. This could attenuate the relative impact of cancer stage on AF risk in this group. In terms of sex, prior studies have suggested that female sex is associated with a younger age and more aggressive tumor biology in advanced gastric cancer patients [15,16]. These sex-related biological differences of advanced gastric cancer, along with the pro-inflammatory effect of female sex hormones (e.g., estrogen), may lead to heightened systemic inflammation and, in turn, may elevate the risk of AF in the female subgroup. Regarding age, younger patients are more likely to receive intensive treatment regimens, including higher doses of chemotherapy or combination therapies. Although our multivariable model adjusted for initial treatment modalities, detailed information on specific chemotherapy agents and dosing was not available in the CPLD database. Such treatment-related differences may have contributed to the increased incidence of AF observed among younger patients with advanced-stage gastric cancer.

Differences in treatment according to cancer stage may also represent an important confounding factor. In particular, chemotherapeutic agents commonly used as first-line therapy in distant-stage gastric cancer, such as capecitabine and 5-fluorouracil, have been associated with cardiovascular complications [17]. While coronary vasospasm leading to myocardial ischemia is the most frequently reported cardiotoxicity, arrhythmias, including atrial fibrillation (AF), have also been observed with these agents [18,19,20]. To account for such a cardiotoxicity of chemotherapeutic agents in advanced gastric cancer patients, this study utilized a multivariable Cox proportional hazards model adjusted for initial treatments within 4 months (Model 5), which still showed a significant relationship between cancer stage and AF risk. However, the extent of the increase was less prominent than that in Model 4, which did not adjust for the initial treatments. This suggests that the initial treatment may have partially contributed to the elevated risk of AF in advanced GC.

Some studies suggest that the risk of AF is highest early after cancer diagnosis and is no longer significant 5 years after diagnosis [6,7]. A meta-analysis revealed that AF rates in cancer survivals who have survived longer than 12 months were not significantly higher compared to the general population, suggesting a time-dependent association between cancer and AF [21]. In line with these results, in this study, patients diagnosed within 5 years showed a stronger correlation between cancer stage and AF risk. This can be explained by more pronounced systemic inflammation and cancer-associated complications shortly after diagnosis. Survival and surveillance biases may also be present, as patients with advanced-stage cancer in poor general condition, who are more likely to develop AF, are less likely to survive beyond 5 years, whereas healthy patients with early-stage cancer tend to have fewer visits to healthcare facilities after 5 years. Nevertheless, cancer stage remained a significant risk factor for AF even after 5 years, highlighting the fact that advanced cancer is a risk factor for AF, regardless of the time of diagnosis.

Currently, screening for AF is recommended by routine heart rhythm assessment during healthcare contact in individuals aged ≥ 65 [22,23]. Moreover, risk prediction models that incorporate conventional risk factors—such as age, smoking status, hypertension, and coronary artery disease—are commonly used for stratifying AF risk [24,25,26]. However, there is limited evidence supporting AF screening specifically in cancer populations. The findings of our study underscore the potential importance of AF screening in patients with advanced gastric cancer, as they may be at increased risk for cardiovascular complications. Furthermore, our results suggest that cancer stage may serve as an independent risk factor for AF and thus could be considered as an additional parameter in future risk prediction models tailored for cancer patients.

There are several limitations to this study. One limitation of our study was that data on biomarkers related to systemic inflammation and metabolic dysregulation were not available. Longitudinal measurements of such markers during follow-up could have provided a more detailed mechanistic insight into the relationship between cancer progression and atrial fibrillation (AF). Moreover, the treatment data were limited to initial therapies administered within the first four months after diagnosis. As a result, any subsequent changes, delays, or additions to treatment could not be accounted for in the multivariable Cox proportional hazards models, potentially introducing unmeasured confounding. Moreover, the ICD-O-3 code C16 used to identify gastric cancer patients includes both gastric carcinoma and gastric neuroendocrine tumors (NETs), which may have introduced a potential source of bias. However, the majority of gastric cancers are carcinomas, and gastric NETs have been reported to account for only 0.3% to 1.8% of all gastric cancers [27]. Therefore, the impact of this subgroup on the overall findings is considered minimal. Additionally, given the high mortality associated with advanced gastric cancer, the cumulative incidence of AF may be influenced by competing risks, such as cancer-related death. This may have led to an underestimation of AF’s incidence. Finally, as the Korean population is predominantly of a single ethnic background, the generalizability of our findings may be limited. In particular, the epidemiology and biologic behavior of gastric cancer differs between Asian and non-Asian populations [28,29,30], highlighting the need for the further validation of our results in more ethnically diverse cohorts.

## 5. Conclusions

This study utilized a comprehensive national database to demonstrate a significant association between GC stage and new-onset AF, with higher hazard ratios in advanced stages. This association was more pronounced in the younger, non-hypertensive, and female subgroups. These results highlight the potential clinical importance of vigilant monitoring for AF in patients with advanced-stage GC, particularly in those without traditional AF risk factors.

## Figures and Tables

**Table 1:** Baseline characteristics of the study population.

	No. (%)				
	Total Population (N = 211,500)	SEER Summary Stage			*p*-Value
		Localized (N = 141,728)	Regional (N = 45,656)	Distant (N = 24,116)	
Age at diagnosis (years)					<0.0001
30–39	5682 (2.69)	3171 (2.24)	1437 (3.15)	1074 (4.45)	
40–64	103,642 (49)	71,489 (50.44)	20,968 (45.93)	11,185 (46.38)	
≥65	102,176 (48.31)	67,068 (47.32)	23,251 (50.93)	11,857 (49.17)	
Sex					0.0002
Male	143,244 (67.73)	96,397 (68.02)	30,606 (67.04)	16,241 (67.35)	
Female	68,256 (32.27)	45,331 (31.98)	15,050 (32.96)	7875 (32.65)	
Initial treatment					
Surgery	174,298 (82.41)	129,859 (91.63)	38,753 (84.88)	5686 (23.58)	<0.0001
Chemotherapy	42,532 (20.11)	6972 (4.92)	20,401 (44.68)	15,159 (62.86)	<0.0001
Radiotherapy	1628 (0.77)	180 (0.13)	606 (1.33)	842 (3.49)	<0.0001
Immunotherapy or hormone therapy	214 (0.1)	69 (0.05)	43 (0.09)	102 (0.42)	<0.0001
Year of diagnosis (year)					
2012–2013	53,749 (25.41)	34,888 (24.62)	12,598 (27.59)	6263 (25.97)	
2014–2015	52,526 (24.83)	34,878 (24.61)	11,576 (25.35)	6072 (25.18)	
2016–2017	53,845 (25.46)	36,683 (25.88)	11,115 (24.35)	6047 (25.07)	
2018–2019	51,380 (24.29)	35,279 (24.89)	10,367 (22.71)	5734 (23.78)	
Comorbidities					
Diabetes mellitus	43,637 (20.63)	28,880 (20.38)	9707 (21.26)	5050 (20.94)	<0.0001
Hypertension	94,894 (44.87)	63,970 (45.14)	20,540 (44.99)	10,384 (43.06)	<0.0001
Dyslipidemia	57,169 (27.03)	41,558 (29.32)	10,787 (23.63)	4824 (20)	<0.0001
Income level					<0.0001
Medical aid + 1st and 2nd decile	41,181 (19.47)	26,206 (18.49)	9671 (21.18)	5304 (21.99)	
3rd to 8th decile	105,975 (50.11)	70,187 (49.52)	23,400 (51.25)	12,388 (51.37)	
9th and 10th decile	64,344 (30.42)	45,335 (31.99)	12,585 (27.56)	6424 (26.64)	
Residential area, urban	91,103 (43.07)	61,443 (43.35)	19,330 (42.34)	10,330 (42.83)	0.0005
Follow-up duration (years)	3.89 ± 2.58	4.49 ± 2.41	3.47 ± 2.55	1.12 ± 1.37	<0.0001

SEER—Surveillance, Epidemiology, and End Results; BMI—body mass index. Continuous variables (mean ± standard deviation); categorical variables, numbers (percentage).

**Table 2:** Risk of atrial fibrillation according to the SEER stage at the time of diagnosis of gastric cancer.

SEER Stage	N	Event	IR ^a^	HR (95% CI)				
				Model 1	Model 2	Model 3	Model 4	Model 5
Localized	141,728	4765	7.49	1 (Ref.)	1 (Ref.)	1 (Ref.)	1 (Ref.)	1 (Ref.)
Regional	45,656	1754	11.06	1.39 (1.32–1.47)	1.38 (1.30–1.45)	1.38 (1.30–1.45)	1.39 (1.32–1.47)	1.32 (1.25–1.41)
Distant	24,116	747	27.73	2.29 (2.11–2.48)	2.33 (2.15–2.52)	2.33 (2.15–2.52)	2.39 (2.20–2.59)	2.00 (1.81–2.22)
*p*-value								

SEER: Surveillance, Epidemiology, and End Results; IR: incidence rate; HR: hazard ratio; CI: confidence interval. Model 1: Not adjusted. Model 2: Adjusted for age, sex. Model 3: Model 2 + adjusted for income level, residential area. Model 4: Model 3 + adjusted for diabetes mellitus, hypertension, dyslipidemia. Model 5: Model 4 + Adjusted for initial therapy. ^a^ Number of cases per 1000 person-years.

**Table 3:** Subgroup analysis.

		SEER Stage	N	Event	IR ^a^	Adjusted HR ^b^ (95% CI)	*p* for Interaction
Age	30–64	Localized	74,660	1281	3.59	1 (Ref.)	<0.0001
		Regional	22,405	484	5.35	1.46 (1.30–1.63)	
		Distant	12,259	302	18.29	3.20 (2.77–3.70)	
	≥65	Localized	67,068	3484	12.48	1 (Ref.)	
		Regional	23,251	1270	18.63	1.30 (1.21–1.39)	
		Distant	11,857	445	42.71	1.63 (1.45–1.83)	
Sex	Male	Localized	96,397	3540	8.21	1 (Ref.)	0.0016
		Regional	30,606	1244	11.76	1.28 (1.10–1.38)	
		Distant	16,241	513	28.14	1.84 (1.64–2.06)	
	Female	Localized	45,331	1225	5.96	1 (Ref.)	
		Regional	15,050	510	9.65	1.43 (1.28–1.59)	
		Distant	7875	234	26.88	2.47 (2.12–2.87)	
Year of diagnosis	2012–2013	Localized	34,888	1757	7.17	1 (Ref.)	<0.0001
		Regional	12,598	617	9.79	1.27 (1.15–1.39)	
		Distant	6263	185	22.47	1.78 (1.51–2.10)	
	2014–2015	Localized	34,878	1388	7.37	1 (Ref.)	
		Regional	11,576	453	9.84	1.21 (1.08–1.35)	
		Distant	6072	163	23.16	1.68 (1.41–2.00)	
	2016–2017	Localized	36,683	1010	7.41	1 (Ref.)	
		Regional	11,115	369	11.33	1.34 (1.18–1.51)	
		Distant	6047	187	27.89	2.10 (1.77–2.49)	
	2018–2019	Localized	35,279	610	9.14	1 (Ref.)	
		Regional	10,367	315	18.52	1.72 (1.50–1.98)	
		Distant	5734	212	42.76	2.79 (2.36–3.31)	
History of diabetes mellitus	No	Localized	112,848	3512	6.80	1 (Ref.)	0.4644
		Regional	35,949	1261	9.79	1.30 (1.22–1.40)	
		Distant	19,066	552	25.20	2.04 (1.82–2.28)	
	Yes	Localized	28,880	1253	10.43	1 (Ref.)	
		Regional	9707	493	16.52	1.38 (1.24–1.53)	
		Distant	5050	195	38.77	1.92 (1.63–2.26)	
History of hypertension	No	Localized	77,758	1592	4.40	1 (Ref.)	0.0021
		Regional	25,116	633	6.77	1.40 (1.27–1.54)	
		Distant	13,732	313	18.99	2.38 (2.08–2.74)	
	Yes	Localized	63,970	3173	11.57	1 (Ref.)	
		Regional	20,540	1121	17.21	1.29 (1.2–1.39)	
		Distant	10,384	434	41.52	1.81 (1.61–2.04)	
History of dysplipidemia	No	Localized	100,170	3022	6.50	1 (Ref.)	0.3318
		Regional	34,869	1196	9.59	1.31 (1.22–1.41)	
		Distant	19,292	548	25.21	2.07 (1.85–2.31)	
	Yes	Localized	41,558	1743	10.16	1 (Ref.)	
		Regional	10,787	558	16.49	1.35 (1.23–1.49)	
		Distant	4824	199	38.32	1.85 (1.57–2.17)	

IR: incidence rate; HR: hazard ratio; CI: confidence interval; ^a^ Number of cases per 1000 person-years; ^b^ Adjusted for age, sex, income, residential area, diabetes mellitus, hypertension, dyslipidemia, initial treatment.

## Data Availability

Data, analytic methods, and study materials will be available to other researchers upon reasonable request.

## Supplementary Materials

The following supporting information can be downloaded at: https://www.mdpi.com/article/10.3390/cancers17122054/s1, Table S1: Operational definitions of comorbidities.
