# Supplementary material for: Atrial Fibrillation Risk in Relation to the Clinical Staging of Gastric Cancer: A Nationwide Population-Based Cohort Study
Source: Cancers (Basel). 2025 Jun 19;17(12):2054. doi: 10.3390/cancers17122054 (PMC12190469; doi:10.3390/cancers17122054)
Supplement: Supplementary file 1 [file cancers-17-02054-s001.zip › cancers-3661690-supplementary.pdf]

Supplementary Table S1. Operational definitions of comorbidities

Comorbidities	ICD-10 Codes and Definition	Diagnostic Definition
Diabetes mellitus	E11-E14; and minimum one prescription of anti-diabetic drugs (sulfonylurea, metformin, meglitinide, thiazolidinedione, dipeptidyl peptidase-4 inhibitor, α-glucosidase inhibitor, and insulin).	Admission≥1 or outpatient department≥1
	Or fasting glucose level ≥ 126 mg/dL	Index health examination
Hypertension	I10-I13, I15; and minimum one prescription of anti-hypertensive medication (thiazide, loop diuretic, aldosterone antagonist, alpha/beta-blocker, calcium channel blocker, angiotensin-converting enzyme inhibitor, and angiotensin II receptor blocker).	Admission≥1 or outpatient department≥2
	Or systolic/diastolic blood pressure ≥ 140/90 mmHg	Index health examination
Dyslipidemia	E78.	Admission or outpatient department≥1
	Or total cholesterol ≥ 240 mg/dL	Index health examination
